# Compliance in the deep and superficial conduit veins of the nonexercising arm is unaffected by short‐term exercise

**DOI:** 10.14814/phy2.13724

**Published:** 2018-06-04

**Authors:** Anna Oue, Tomoko Sadamoto

**Affiliations:** ^1^ Faculty of Food and Nutritional Sciences Toyo University Gunma Japan; ^2^ Research Institute of Physical Fitness Japan Women's College of Physical Education Tokyo Japan

**Keywords:** Cuff deflation protocol, sympathoexcitation, ultrasonography, venoconstriction

## Abstract

The effects of short‐term dynamic and static exercise on compliance (CPL) in a single conduit vein in the nonexercising limb are not fully understood, although prolonged cycling exercise was found to produce a significant reduction of CPL in the veins. In this study, we investigated the cross‐sectional area (CSA) and CPL in the brachial (deep) and basilic (superficial) veins of the nonexercising arm in 14 participants who performed a 5‐min cycling exercise at 35% and 70% of peak oxygen uptake (study 1) and in 11 participants who performed a 2‐min static handgrip exercise at 30% of maximal voluntary contraction (study 2). The CSA in the deep and superficial veins at rest and during the final minute of exercise was measured by high‐resolution ultrasonography during a short‐duration cuff deflation protocol. The CPL in each vein was calculated as the numerical derivative of the cuff pressure and CSA curve. During short‐term dynamic and static exercise, there was no change in CPL in either vein, but there was a decrease in CSA in both veins. The simultaneous findings of unchanged CPL and decreased CSA suggest that CPL during short‐term exercise are independently controlled by the mechanisms responsible for exercise‐induced sympathoexcitation in both single veins. Thus, short‐term exercise does not alter CPL in both conduit superficial and deep veins in nonexercising upper arm.

## Introduction

The veins have high compliance (CPL), so the venous component of the cardiovascular system plays an important role as a blood reservoir, containing approximately 70% of the total blood volume at rest. Physiologic stressors, such as exercise, orthostatic stress, and exposure to heat, change venous capacitance (Drees and Rothe 1974; Greenway et al. 1985), CPL, or both (Shoukas et al. 1981; Monahan and Ray 2004), which could assist the shift of blood from veins to the heart and maintain central blood volume and blood pressure (Rothe 1983).

The CPL in a single conduit vein during exercise has not been adequately examined, although we previously found that prolonged dynamic cycling exercise produced significantly decreased CPL and cross‐sectional area (CSA) in a single conduit vein in the nonexercising upper arm (Oue et al. 2017). This was the first report of reduction in CPL at the level of a single conduit vein during exercise, despite ample data for venous CPL in a whole limb measured by venous occlusion plethysmography (VOP) (Freeman et al. 2002; Monahan and Ray 2004; Hernandez and Franke 2005; Young et al. 2006; Delaney et al. 2008). From the simultaneous reductions in CSA and CPL during prolonged exercise seen in our previous study (Oue et al. 2017), it was thought that the decrease in CPL was induced by exercise‐induced sympathoexcitation (venoconstriction). However, the prolonged exercise in the previous study (Oue et al. 2017) was accompanied by a significant increase in core temperature. Since the elevation of core temperature during passive heating was known to be one of conceivable factors that altered CPL (Tripathi et al. 1984), it was unclear in the previous study whether exercise‐induced sympathoexcitation per se or the concomitant increase in core temperature caused the reduction in CPL during prolonged exercise.

To understand better the CPL during exercise, it was necessary to examine whether exercise‐induced sympathoexcitaion that does not produce a significant elevation in core temperature leads to a reduction in CPL. Given that core temperature does not change during 5‐min dynamic cycling exercise or during a 2‐min static handgrip exercise (SHG) (Smolander et al. 1991; Crandall et al. 1998; Kondo et al. 1998), these forms of short‐term exercise would be suitable for investigating the cause of reduction of CPL during exercise. In addition, dynamic cycling exercise with large muscle mass produces a greater activation of sympathetic nerve activity (SNA) and plasma norepinephrine concentration than SHG with small muscle mass (Davies et al. 1974; Blomqvist et al. 1981), and that high‐intensity exercise induces a greater SNA and norepinephrine than low‐intensity exercise (Lewis et al. 1983, 1985). Since the degree of venconstriction in nonexercising limb during short‐term exercise depended on the magnitude of exercise‐induced sympathoexcitation (Rowell et al. 1971), venous CPL is also expected to be influenced by the amplitude of activated SNA during exercise. Thus, short‐term exercise with different modes and intensities would be suitable for determining the effect of exercise‐induced sympathoexcitation on CPL in the vein.

The large conduit veins in a limb are roughly divided into two different types, deep and superficial. For example, the veins in the arm are separated into deep veins (e.g., the brachial vein), which run along the brachial artery beneath the muscle tissue, and superficial veins (e.g., the basilic and cephalic veins), which run beneath the cutaneous tissue (Roddie et al. 1956). The deep veins contain less sympathetic innervation than the superficial veins (Abdel‐Sayed et al. 1970). Given that the superficial and deep conduit veins are not uniform anatomically or functionally, the responses to sympathoexcitation during short‐term dynamic and static exercise might be different, thereby leading to different CPL responses during exercise.

The aim of this study was to investigate the effects of a 5‐min dynamic cycling exercise at low and high intensities on CPL (study 1) and the effects of 2‐min SHG on CPL (study 2) in the superficial (basilic) vein and deep (brachial) vein in the nonexercising upper limb. To assess CPL in a single conduit vein, the CSA of the deep vein and that of the superficial vein were measured by high‐resolution ultrasonography during the short‐duration cuff deflation protocol devised by Halliwill et al. (1999). This protocol was chosen because (1) the methodology of the short cuff deflation protocol allows an overall pressure–CSA curve to be generated without the assumption that resting venous pressure is zero, as is assumed when a cuff inflation protocol is used; (2) venous CPL measurements during cuff deflation are minimally influenced by capillary filtration when the duration of occlusion is short (4–8 min); and (3) the methodology can be used during sympathoexcitatory maneuvers. Ultrasonography also allows the response of a single vein to be assessed. A single vein seems to be less influenced by shifts of interstitial fluid, so measurement of CPL by ultrasonography would reflect the change in CPL in the vein itself rather than that measured by VOP (de Groot et al. 2005). Moreover, several other research groups have measured the CSA and CPL of a single vein at rest using ultrasonography (de Groot et al. 2005; Young et al. 2008; Zachrisson et al. 2011; Leinan et al. 2015, 2016; Oue et al. 2017).

## Materials and Methods

### Subjects

Participants were 25 healthy volunteers (11 men, 14 women) who were instructed not to ingest caffeine for 24 h or food for 2 h before each experiment. Informed consent was obtained from all participants after they had received an explanation of the purpose, procedures, and risks of the study. The study was approved by the Human Ethics Committee of the Japan Women's College of Physical Education and was conducted in accordance with the tenets of the Declaration of Helsinki.

### Study 1

In study 1, we investigated the response of CPL in the superficial vein and deep vein of a resting upper limb during cycling exercise. Fourteen healthy volunteers (4 men, 10 women) participated in this study. Their mean (±SD) age, height, weight, and peak oxygen uptake ($\dot{V}$O_2peak_) were 21.4 ± 2.7 years, 163.6 ± 8.8 cm, 54.6 ± 6.9 kg, and 38.6 ± 4.4 mL min^‐1^ kg^‐1^, respectively. All participants reported to the laboratory on three occasions.

$\dot{V}$O_2peak_ was determined during incremental cycling exercise during the first session and CPL was measured in the superficial or deep vein during the second and third sessions. Each experimental session was performed in a thermoneutral environment (27.1°C ± 0.4°C) to evaluate CPL in each vein at rest and during dynamic exercise. After participants had rested in a seated position on a cycle ergometer (StrengthErg, Mitsubishi Electric Engineering, Co., Ltd., Tokyo, Japan) for at least 30 min, venous CPL was measured at rest (REST1). Venous CPL was then assessed during 5 min of cycling exercise at low intensity (35%$\dot{V}$O_2peak_; EX35%) and at high intensity (70%$\dot{V}$O_2peak_; EX70%), with each assessment separated by a 15–20‐min interval of quiet rest. In addition, exercise at both intensities was performed randomly. Measurements of each vein were randomized between the second and third sessions.

### Study 2

In study 2, we investigated the response of CPL in the superficial vein and deep vein of a resting upper limb during SHG. Eleven healthy volunteers (7 men, 4 women) with mean age, height, and weight of 21.9 ± 2.0 years, 166.9 ± 9.2 cm, and 57.1 ± 7.3 kg, respectively, participated in this study. They reported to the laboratory on two occasions, one for measurement of CPL in the superficial vein and the other for measurement of CPL in the deep vein. Each experiment was performed in a thermoneutral environment (26.2°C ± 0.4°C) to evaluate CPL in each vein at rest and during SHG as follows. After participant had rested in the supine position for at least 20 min, venous CPL in the left arm was measured in the resting condition (REST2) and again during SHG at 30% maximal voluntary contraction for 2 min. During all experiments, participants were required to maintain a respiratory rate of 10–15 breaths per min guided by a metronome. Measurements of each vein were randomized between the first and second sessions.

### Peak oxygen uptake

In study 1, $\dot{V}$O_2peak_ was determined by an incremental load exercise protocol (5–20 W min^‐1^, pedaling frequency 60 rpm) using a cycle ergometer (StrengthErg, Mitsubishi Electric Engineering). During the incremental load exercise, ventilatory and gas exchange parameters were measured breath‐by‐breath using a mass spectrometer (ARCO‐1000, Arco System, Chiba, Japan). At least two of the following criteria were used to evaluate $\dot{V}$O_2peak_: maximal or near‐maximal values for rating of perceived exertion; a respiratory gas exchange ratio >1.05; heart rate (HR) within 10 bpm of the age‐predicted maximal HR; and inability to maintain a pedaling frequency of 50 rpm.

### Venous CSA and CPL

The basilic vein was selected as the superficial vein and the brachial vein as the deep vein. For CSA measurements under all conditions in study 1 and study 2, the venous collecting cuff was wrapped around the right upper arm and inflated to 60 mmHg for 8 min. The cuff pressure was then manually reduced over a 1‐min period at a rate of 1 mmHg sec^‐1^ from 60 mmHg to 0 mmHg according to the cuff deflation protocol described previously (Halliwill et al. 1999). In study 1, during EX35% and EX70%, participants performed cycling exercise at 35%$\dot{V}$O_2peak_ or 70%$\dot{V}$O_2peak_ starting after 4 min of cuff inflation and stopping at the end of deflation (total exercise time, 5 min). In study 2, participants performed SHG starting after 7 min of cuff inflation and stopping at the end of deflation (total exercise time, 2 min). During cuff deflation, transverse scans of the deep and superficial veins were obtained 5–6 cm proximal from the right elbow in the resting arm by high‐resolution B‐mode ultrasonography (Vivid e, GE Healthcare, Japan) equipped with a linear‐array transducer with a mean transmission frequency of 8.7 MHz. The CSA was calculated by manually tracing the edge of the transverse venous image at 4‐sec intervals (4 mmHg). The relationship between cuff pressure and CSA (i.e., the pressure–CSA curve) was generated from the datum points between 60 mmHg and 10 mmHg during the cuff deflation protocol. To avoid any a priori assumption regarding the pressure (P)–CSA curve and obtain the physiologic CPL curve, CPL was calculated as the numerical derivative of each pair of pressure–CSA datum points with the following equation (Freeman et al. 2002; Oue et al. 2017).

$$\mathrm{CP}L_{\mathrm{Pi}}\;=\;\frac{\mathrm{CS}A_{\mathrm{Pi}}\;-\mathrm{CS}A_{Pi-1}}{P_{i}\;-P_{i-1}}\;\;\text{where}\;1\;\leq i\;\leq 60$$

The coefficient of variation (CV) for repeated measurements of baseline CSA was 4.2 ± 0.6% in the superficial vein and 5.8 ± 0.9% in the deep vein in study 1 and 2.2 ± 0.3% in the superficial vein and 6.7 ± 0.8% in the deep vein in study 2.

### Circulatory variables

In both studies, mean arterial pressure (MAP) was measured noninvasively using photoelectric plethysmography with a Finometer (Finapres Medical Systems BV, Arnhem, Netherlands), which was calibrated with upper cuff and height adjustment. Furthermore, HR and stroke volume, hence cardiac output (CO), were determined from the blood pressure waveform using the Modelflow software program (BeatScope 1.1, Finapres Medical Systems BV, Amhem, Netherlands), which takes into account the participant's sex, age, height, and weight.

### Statistical analysis

In study 1, MAP, HR, and CO values from 1 to 3 min were averaged during cuff inflation as the baseline data and during cuff deflation as the exercise data. In study 2, the same values from 5 to 7 min during cuff inflation were averaged as the baseline data and during cuff deflation as the exercise data. The relative change in SV and CO during cuff deflation was then calculated from the baseline data as a percentage. In both studies, MAP, HR, and CO values were similar in the deep and superficial veins in all participants, so the average values on two occasions were considered representative for each variable.

To compare the changes with cuff pressure between rest and exercise, two‐way analysis of variance (ANOVA) with repeated measures was applied to the CSA and CPL obtained by a cuff pressure of 10–60 mmHg under each condition (rest and exercise), using cuff pressure and condition as fixed factors. If a main effect of condition and/or interaction was detected, post hoc analysis with a Bonferroni test was performed every 4 mmHg in study 1. If a main effect of condition and/or interaction was detected, post hoc analysis with a paired *t*‐test was performed every 4 mmHg in study 2. Intraclass correlation coefficients and the CV were used to determine the reproducibility of the CSA and CPL in the superficial and deep veins as assessed by ultrasonography. The CV was calculated as the SD divided by the mean and multiplied by 100%. CVs were determined for the absolute CSA and CPL of each vein at the different cuff pressures. For biological variables, CV < 10% is considered good and CV < 20% is considered acceptable (Scott et al. 1989). Statistical significance was set at *P* < 0.05. All data are presented as the mean ± SE of the mean.

## Results

In study 1, MAP, HR, and CO increased significantly during dynamic cycling exercise in accordance with increasing exercise intensity (Table 1). Compared with REST1, the rate of increase in MAP and HR during EX35% was 113.4 ± 2.2% and 153.7 ± 3.1%, respectively, and that in MAP and HR during EX70% was 137.8 ± 4.3% and 226.5 ± 6.0%, respectively.

**Table 1:** Mean arterial pressure (MAP), heart rate (HR), and cardiac output (CO) during REST1, EX35%, and EX70%

	REST1	EX35%	EX70%
MAP, mmHg	80 ± 2	90 ± 2^a^	110 ± 3^a^^,^ b
HR, bpm	68 ± 3	103 ± 2^a^	151 ± 6^a^, ^b^
CO, %	100.9 ± 0.8	196.0 ± 7.1^a^	328.4 ± 15.0^a^^,^ b

Values represent the means ± SE.

a *P* < 0.05, significant difference from REST1.

b *P* < 0.05, significant difference from EX35%.

**Figure 1:**
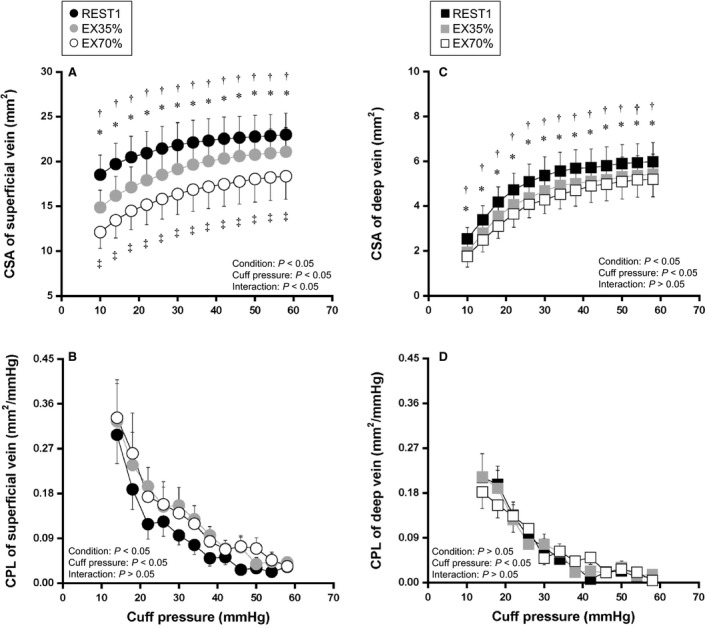
Relationship of cuff pressure–cross‐sectional area (CSA) and cuff pressure–compliance (CPL) of a superficial vein and a deep vein during the resting condition (REST1) and during dynamic cycling exercise at 35%($\dot{V}$)O_2peak_ (EX35%) and 70%($\dot{V}$)O_2peak_ (EX70%). Values are mean ± SE. *P < 0.05, significant difference between REST1 and EX35%, †*P* < 0.05, significant difference between REST1 and EX70%, ^‡^ *P* < 0.05, significant difference between EX35% and EX70%.

Figure 1 shows the effects of dynamic exercise on CSA (CSA_sup_ and CSA_deep_) and CPL (CPL_sup_ and CPL_deep_) in each vein. In both veins, the cuff pressure–CSA curve shifted downwards during dynamic exercise compared with the resting condition (Fig. 1A and C). ANOVA yielded a significant condition effect (*P* < 0.01), and the post hoc test showed a significant difference in mean CSA_sup_ between REST1 and EX35%, between REST1 and EX70%, and between EX35% and EX70% at all cuff pressures (*P* < 0.05), as well as a significant difference in mean CSA_deep_ between REST1 and EX35% and between REST1 and EX70% at all cuff pressures (*P* < 0.05). ANOVA yielded a significant effect of condition (*P* < 0.05) on the cuff pressure–CPL_sup_ relationship (Fig. 1B), but a post hoc test failed to show significant difference in mean CPL_sup_ between rest and exercise. CPL_deep_ was not altered by dynamic exercise (Fig. 1D).

In study 2, SHG caused significant increases in MAP, HR, and CO (Table 2). Compared with REST2, the rate of increase in MAP and HR in SHG was 119.4 ± 1.8% and 115.8 ± 2.0%, respectively.

**Table 2:** Mean arterial pressure (MAP), heart rate (HR), and cardiac output (CO) during REST2, and SHG

	REST2	SHG
MAP, mmHg	78 ± 2	93 ± 3^a^
HR, bpm	64 ± 2	74 ± 3^a^
CO, %	99.2 ± 0.7	111.9 ± 4.4^a^

Values represent the means ± SE.

a *P* < 0.05, significant difference between REST2 and SHG.

**Figure 2:**
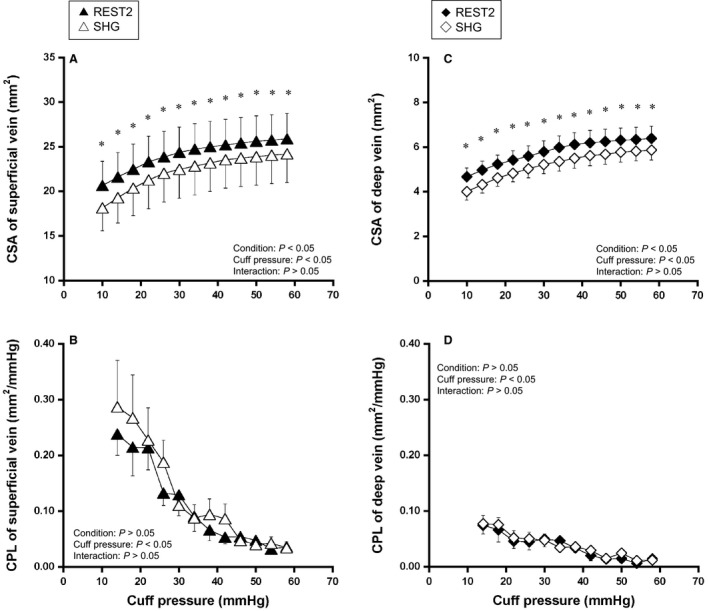
Relationship of cuff pressure–cross‐sectional area (CSA) and cuff pressure–compliance (CPL) of a superficial vein and a deep vein during the resting condition (REST2) and static handgrip exercise (SHG). Values are mean ± SE.*P < 0.05, significant difference between REST2 and SHG.

Figure 2 shows the effects of static exercise on CSA and CPL in each vein. Both CSA_sup_ and CSA_deep_ curves were decreased during SHG compared with REST2 (*P* < 0.05, Fig. 2A and C). However, cuff pressure–CPL_sup_ relationship and cuff pressure–CPL_deep_ relationship was not changed by SHG (Fig. 2B and D).

## Discussion

The primary findings of this study were that dynamic exercise at low and high intensities and static exercise induced parallel downward shifts of the cuff pressure–CSA curve in both the superficial and deep veins and that neither dynamic exercise nor static exercise changed the cuff pressure–CPL relationship in either vein. The simultaneous occurrence of reduced CSA and unchanged CPL suggests that CPL during short‐term exercise were independently controlled by the mechanisms responsible for the reduced CSA, that is, exercise‐induced sympathoexcitation, in conduit superficial and deep veins in the nonexercising limb.

### Measurement of venous CPL using ultrasonography and a short cuff deflation protocol

Our rationale for investigating the effects of exercise on CPL at the single vein level using ultrasonography was that a single vein is less likely to be influenced by the vascular changes that typically occur during exercise (e.g., changes in muscle mass and interstitial fluid shifts). Therefore, measurement of CPL at the single vein level would reflect the change in CPL in the vein itself rather than changes in CPL at the whole limb level when measured by VOP (de Groot et al. 2005). We used the short‐duration cuff deflation protocol validated by Halliwill et al. (1999). This protocol considers that resting venous pressure is not equal to zero and therefore allows assessment of venous CPL during short‐term sympathoexcitatory maneuvers with small hysteresis. The effects of sympathoexcitation (cold pressor test and postexercise muscle ischemia) on venous CPL have already been investigated using a short cuff deflation protocol (Monahan and Ray 2004; Young et al. 2006, 2008; Delaney et al. 2008). In addition, we assessed reproducibility to confirm the validity of using ultrasonography during a short cuff deflation protocol. The CV for CSA at baseline and during exercise (1.2–6.0% in the superficial vein and 0.5–4.8% in the deep vein) was less than 10% and similar to our previously reported data for the basilic vein (Ooue et al. 2013) and other studies of the popliteal vein (de Groot et al. 2005). In addition, the intraclass correlation coefficients for the CSA and CPL of the two veins at baseline and during exercise were more than 0.93, respectively, which is consistent with another study reporting an intraclass correlation coefficient of 0.92 for baseline venous CPL assessed by ultrasonography (Young et al. 2008). Thus, using ultrasonography during a short cuff deflation protocol seems to be appropriate for investigating the effects of exercise on CPL at the level of the vein itself.

### Decreased CSA in the superficial and deep veins during dynamic and static exercise

In this study, both dynamic and static exercise produced a downward shift of the cuff pressure–CSA curves for the superficial and deep veins from the resting level. The reduction in CSA during dynamic and static exercise was similarly observed in our previous studies using the same ultrasonography technique to assess a basilic vein during cycling exercise (Ooue et al. 2008) and static exercise (Ooue et al. 2012, 2013). This is also in line with earlier research assessing venous capacitance of a whole limb by VOP during dynamic exercise (Bevegård and Shepherd 1965, 1966; Zelis and Mason 1969; Rowell et al. 1971; Duprez et al. 1992) and the change in venous pressure as an index of venoconstriction during static exercise (Lorentsen 1975). The reduced CSA during exercise in the present study was probably attributable to exercise‐induced sympathoexcitation via neural innervation from central command (Ooue et al. 2012) and from exercising muscle (mechanoreceptors and metaboreceptors) (Duprez et al. 1992; Ooue et al. 2013) and/or an indirect action of circulating norepinephrine, given that the peripheral venous system is richly innervated by sympathetic fibers (Abdel‐Sayed et al. 1970; Vanhoutte and Lorenz 1970; Streeten 1990; Linder et al. 1996). Indeed, higher loads of dynamic exercise (up to 70–75% of $\dot{V}$O_2peak_) targeting large muscle groups, such as cycling, are known to bring about significant increases in cutaneous vasoconstriction (Taylor et al. 1988, 1990; Johnson 1992; Yanagimoto et al. 2003), SNA in muscle (Saito et al. 1993; Saito and Nakamura 1995), and plasma norepinephrine concentrations (Christensen and Brandsborg 1973; Lehmann et al. 1985; Mazzeo and Marshall 1989). In addition, SHG has been reported to induce an increase in SNA in muscle (Saito et al. 1986, 1990; Seals 1989). Based on these considerations, the decrease in CSA in the superficial and deep veins in our study was probably caused by exercise‐induced sympathoexcitation. However, we cannot exclude the effect of passive collapse and/or depression in response to the decrease in pressure (Öberg 1967; Noble et al. 1998) on the decreased CSA of these veins.

### Unchanged CPL in both superficial and deep veins with simultaneous reduction of CSA during dynamic and static exercise

From the simultaneous reductions in CSA and CPL during prolonged exercise in our previous study (Oue et al. 2017), we hypothesized that a decrease in CSA would induce a parallel decrease in CPL probably due to exercise‐induced sympathoexcitation (venoconstriction). Similarly, we hypothesized that CPL during short‐term exercise would also vary according to different modes and intensity of exercise (Lewis et al. 1983, 1985). The increases in HR, MAP, and CO during exercise in the present data varied between EX35%, EX70%, and SHG in parallel with the decrease in CSA indicating that different levels of sympathoexcitation were actually provided in the present experiments. However, there was no significant change in CPL during short‐term dynamic or static exercise from the resting level, and the CPL was almost identical even though exercise mode and intensity were different. Based on these results, it seems likely that the exercise‐induced sympathoexcitation was not an essential determinant for CPL during short‐term exercise in conduit deep and superficial veins. One possible explanation for the unchanged CPL is that the sympathetic venoconstriction induced by short‐term exercise was not sufficient to alter the wall tension in either conduit vein in the nonexercising limbs. This finding is consistent with previous studies, using VOP, that sympathoexcitation during postexercise muscle ischemia (Halliwill et al. 1999; Delaney et al. 2008) and after administration of norepinephrine (Greenway et al. 1985) resulted in unchanged CPL but caused decreased venous volume. Another possible explanation is that the reduction in CSA masked or compensated for a possible decrease in CPL induced by sympathetic venoconstriction during short‐term exercise, given that the tension‐length relationship is known to be true in terms of the relationship between venous wall tension and vein diameter (Rothe 1983). From these previous data and our present findings, we speculate that adjustments of CPL during short‐term exercise might be regulated independently of the mechanisms involved in sympathetic venoconstriction.

### Limitations

This study has some limitations. First, we did not control for stages of the menstrual cycle in our female subjects. However, measurements of venous CPL in the superficial and deep veins were separated by an interval of less than 3 days, meaning that the comparison between the veins would have been in the same luteal or follicular phase of the menstrual cycle. Therefore, we believe that lack of adjustment for the menstrual cycle would not have influence our results. Second, we did not measure SNA or venous pressure directly and no mechanistic investigation was performed. Thus, we cannot completely separate the decreased CSA of the veins into active venoconstriction attributable to sympathoexcitation and passive collapse and/or depression caused by decreased pressure. These limitations need to be addressed in future studies.

## Conclusion

In this study, neither short‐term dynamic exercise nor static exercise changed CPL in a superficial vein or a deep vein in spite of a decrease in CSA in both veins. These results suggest that CPL during short‐term exercise are independently controlled by the mechanisms responsible for exercise‐induced sympathoexcitation in both conduit superficial and deep veins in the nonexercising upper arm.

## Conflict of Interest

The authors have no financial conflicts of interest to declare.
